# Lifestyle Intervention Involving Calorie Restriction with or without Aerobic Exercise Training Improves Liver Fat in Adults with Visceral Adiposity

**DOI:** 10.1155/2014/197216

**Published:** 2014-04-17

**Authors:** Eiichi Yoshimura, Hideaki Kumahara, Takuro Tobina, Takuro Matsuda, Makoto Ayabe, Akira Kiyonaga, Keizo Anzai, Yasuki Higaki, Hiroaki Tanaka

**Affiliations:** ^1^Faculty of Sports and Health Science, Fukuoka University, 8-19-1 Nanakuma, Jonan-ku, Fukuoka 814-0180, Japan; ^2^Faculty of Environmental and Symbiotic Sciences, Prefectural University of Kumamoto, 3-1-100 Tsukide, Higashi-ku, Kumamoto 862-8502, Japan; ^3^Faculty of Nutritional Sciences, Nakamura Gakuen University, 5-7-1 Befu, Jonan-ku, Fukuoka 814-0104, Japan; ^4^Faculty of Nursing and Nutrition, Nagasaki University, 1-1-1 Manabino, Nagayo, Nishisonogi-gun, Nagasaki 851-2195, Japan; ^5^Faculty of Computer Science and Systems Engineering, Okayama Prefectural University, 111 Kuboki, Soja-shi, Okayama 719-1197, Japan; ^6^Department of Internal Medicine, Saga University, 5-1-1 Nabeshima, Saga 849-8501, Japan; ^7^Institute for Physical Activity, Fukuoka University, 8-19-1 Nanakuma, Jonan-ku, Fukuoka 814-0180, Japan

## Abstract

*Objective*. To evaluate the effect of calorie restriction-induced weight loss with or without aerobic exercise on liver fat. *Methods*. Thirty-three adults with visceral adiposity were divided into calorie restriction (CR; *n* = 18) or CR and aerobic exercise (CR + Ex; *n* = 15) groups. Target energy intake was 25 kcal/kg of ideal body weight. The CR + Ex group had a targeted exercise time of 300 min/wk or more at lactate threshold intensity for 12 weeks. *Results*. Reductions in body weight (CR, −5.3 ± 0.8 kg; CR + Ex, −5.1 ± 0.7 kg), fat mass (CR, −4.9 ± 0.9 kg; CR + Ex, −4.4 ± 0.6 kg), and visceral fat (CR, −24 ± 5 cm^2^; CR + Ex, −37 ± 5 cm^2^) were not statistically different between groups. Liver fat decreased significantly in both groups, with no difference between groups. Change in maximal oxygen uptake was significantly greater in the CR + Ex group than in the CR group (CR, −0.7 ± 0.7 mL/kg/min; CR + Ex, 2.9 ± 1.0 mL/kg/min). *Conclusion*. Both CR and CR + Ex resulted in an improved reduction in liver fat; however, there was no additive effect of exercise training.

## 1. Introduction

Nonalcoholic fatty liver disease (NAFLD) is recognized as an independent predictor of insulin resistance and cardiovascular disease and is regarded as a liver phenotype for metabolic syndrome [1]. The approach to the treatment of NAFLD is based on weight loss through lifestyle modification involving calorie restriction and regular physical activity [2].

Regular aerobic exercise reduces liver fat despite minimal or no weight loss [3–5]. This is consistent with our findings of a significant reduction in liver fat following aerobic exercise training [6]. Furthermore, weight loss can be achieved by moderate calorie restriction (CR). Previous research has suggested that CR and increased physical activity reduce liver fat when there is a 3–10% weight loss [2].

Excess visceral fat accumulation is widely known as a risk factor for impaired glucose tolerance, decreased insulin sensitivity, and coronary heart disease and is thought to increase the release of free fatty acids and inflammatory adipokines into the portal vein for transport to the liver [7]. In addition, visceral fat accumulation has been found to be associated with liver fat, insulin resistance, and arteriosclerosis risk markers [8, 9]. Although visceral fat, similarly to liver fat, is reduced through moderate weight loss, previous studies have shown that diet plus exercise-induced weight loss may result in preferential decreases in visceral fat compared with diet alone [10, 11]. However, it is still unclear whether calorie restriction-induced weight loss, together with aerobic exercise (CR + Ex), is more effective than CR alone for the reduction of liver fat in adults with visceral adiposity. We hypothesized that the addition of aerobic exercise to calorie restriction-induced weight loss would show additional reductions in liver fat and liver enzymes than calorie restriction alone. The aim of the present study was to evaluate the effect of calorie restriction-induced weight loss, with or without aerobic exercise, on liver fat in adults with visceral adiposity.

## 2. Methods

### 2.1. Study Population

**Figure 1:**
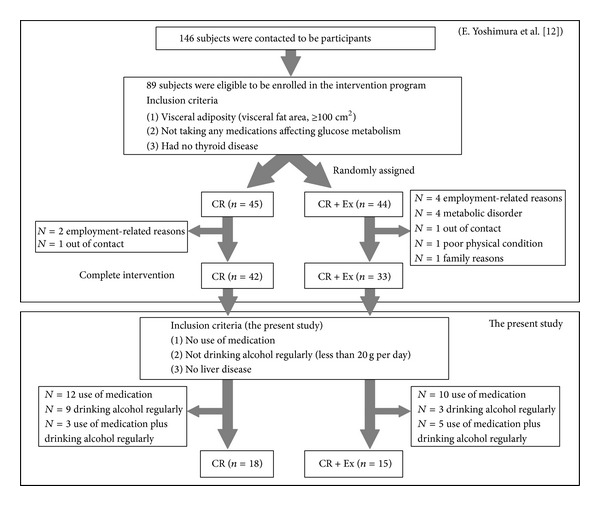
Flow of participants through the study.

The present study was conducted between March 2007 and August 2011 and was approved by the Ethics Committee of Fukuoka University. Males and females aged 40–75 years were recruited by advertisements in newspapers, on television, and on public transportation. Overall, 146 subjects were contacted to participate, and 89 subjects were eligible to be enrolled in the Metabolic Syndrome Prevention/Improvement Intervention Program. The participants enrolled in the study (1) had visceral adiposity (visceral fat area ≥100 cm^2^), (2) were not taking any medications affecting glucose metabolism to avoid potential confounding effects on weight change, and (3) had no thyroid disease. Before baseline measurements, subjects were randomized to receive one of two interventions, with each lasting 12 weeks: diet-induced weight loss through calorie restriction (CR, *n* = 45) or diet-induced weight loss combined with aerobic exercise (CR + Ex, *n* = 44) [12]. Fourteen subjects who did not complete the program left for employment-related reasons, a metabolic disorder or ileus, poor physical condition during the 12-week intervention, or family reasons or were lost to contact [12]. After the intervention program, data of the participants who met the following inclusion criteria were used in the present study: (1) no use of medication; (2) not drinking alcohol regularly (less than 20 g per day); (3) no thyroid disease; and (4) no liver disease, other than fatty liver, based on their past medical histories. Consequently, 42 participants were excluded from this study on the grounds of using medication and drinking alcohol regularly. Finally, 33 men and women were included in the CR group (*n* = 18 [males = 4, females = 14]) or CR + Ex group (*n* = 15 [males = 4, females = 11]). The flow of participants through the study is shown in Figure 1.

### 2.2. Lifestyle Intervention

Daily energy needs were determined by multiplying the participant's ideal body weight (BW; kg), equivalent of BMI 22, by 25. Once a week, the participants received guidance from experienced dieticians (face-to-face), who recommended appropriate daily nutrition via lectures and counseling. The sessions included adjustments of caloric intake and behavioral therapy. Food diaries were reviewed every week, and the participants were given instructions on food intake based on the prescribed energy intake. The participants were instructed to measure their weight daily.

Exercise training consisted of 20 min each of step exercises, bicycle ergometry, and walking or running (60 min per session), three times per week under the supervision of exercise trainers, and a further 120 min/wk (nonsupervised) at home with the aim of achieving at least 300 min/wk of moderate exercise. The International Obesity Task Force (IOTF) [13] and American College of Sports Medicine (ACSM) [14] have indicated that for the prevention of weight gain some degree of moderate-intensity physical activity is required (IOTF: 315 to 420 min/wk, ACSM: 150 to 250 min/wk). Based on these statements, the target value of exercise training was set at 300 min/wk. Exercise intensity was set at a lactate threshold (LT) that was determined from an incremental exercise test on a bicycle ergometer and bench step exercise. The intensity of walking or jogging was controlled through monitoring heart rate (HR) (Polar FT1, Polar Electro, Kempele, Finland) at the LT intensity (determined from the step exercise test). The home exercise sessions (stepping and walking or jogging) were performed using bench step and HR monitor that were provided by the researchers. Each participant recorded all exercise sessions, including the duration (min), type, and HR. These were reviewed every other week to assess exercise adherence. All subjects underwent workload modifications at least 6 weeks after starting the program. The exercise protocol was performed as previously described [6]. The CR + Ex group included aerobic exercise in addition to calorie restriction-induced weight loss. Therefore, the CR + Ex group was supposed to lose more weight than the CR group.

### 2.3. Dietary Records and Physical Activity

To calculate energy intake (in kilocalories) and macronutrient breakdown (fat, protein, and carbohydrate in grams), each participant's self-recorded dietary intake was evaluated before and during the intervention (weeks 10 to 12 of the intervention period). The self-recorded food intake was conducted for a 3-day period including two weekdays and either Saturday or Sunday. All the meals were photographed to increase the accuracy of the measurement.

Physical activity (the number of steps) was assessed using a small uniaxial accelerometer (Lifecorder Ex, Suzuken Co., Ltd., Nagoya, Japan) [15]. The subjects wore the accelerometer, except while sleeping or bathing, for 2 weeks before the intervention and during weeks 10–12 of the intervention. The accelerometer was sealed so that the subjects could not gain access to the physical activity measurements. All of the measured variables were averaged over the last 7 days of the measurement period to assess physical activity under free-living conditions.

### 2.4. Anthropometry Measurements and Body Composition

BW was measured to the nearest 0.01 kg using electronic scales (Shinko Denshi Vibra Co., Ltd., Tokyo, Japan). Height was measured to the nearest 0.1 cm using a stadiometer. Body mass index (BMI: kg/m^2^) was calculated as BW (kg) divided by the square of body height (m^2^). Percent body fat (%FAT), body fat mass (FM), and lean body mass were determined using the underwater weighing method, and body density was estimated after correction for residual air by the O_2_ rebreathing method during underwater weighing. All anthropometric measurements and body composition were conducted after a ≥12-hour fast.

### 2.5. Computed Tomography (CT)

CT protocol was performed as previously described [6]. Visceral fat area (VFA) and subcutaneous fat area (SFA) were assessed at the L4-L5 intervertebral disc space. Liver-to-spleen ratio (L/S) was determined from the calculation of the Hounsfield units (HU) values using CT image analysis software on a Macintosh computer (OsiriX ver. 3.3; OsiriX Foundation, Geneva, Switzerland). The mean HU values of the liver and spleen also were obtained to determine L/S [16]. A lower L/S ratio equates with high liver fat. Conversely, a higher L/S ratio represents low liver fat. All subjects fasted for at least 3 hours but were allowed water, before the CT examination.

### 2.6. Aerobic Fitness

Peak aerobic capacity (VO_2peak_) was determined using an incremental exercise test on a bicycle ergometer (Rehcor; Lode BV, Groningen, The Netherlands). The test was continued until subjective exhaustion was achieved. We assumed that participants had reached maximal oxygen uptake (VO_2max⁡_) when at least two of the following criteria were met: (1) a plateau in VO_2_ with an increase in work load; (2) blood lactate levels ≥8.0 mmol/L; (3) respiratory exchange ratio ≥1.10; (4) HR within 10 beats of the predicted maximum HR; and (5) ratings of perceived exertion ≥19. Respiratory gas analysis was conducted using the mixing chamber method to evaluate the volume of expired air, and the O_2_ and CO_2_ fractions were analyzed by mass spectrometry (ARCO 1000 and 2000, Arco System, Chiba, Japan). Lactic acid was analyzed using the Biosen 5040-lactate analyzer (EKF Diagnostik, Barleban, Germany). To take into account individual differences in BW, oxygen uptake was expressed as kilograms of BW.

### 2.7. Blood Biochemistry

Blood samples were obtained from the antecubital vein in the morning after a 12-hour overnight fast. Serum biochemistry analysis was conducted by SRL Inc. (Tokyo, Japan). The biochemical parameters included interleukin-6 (IL-6), insulin, high-sensitive C-reactive protein (hsCRP), high-molecular-weight adiponectin, tumor necrosis factor- (TNF-) *α*, glucose (a fasting glucose and 2-hour 75 g oral glucose tolerance test [OGTT_120min_-Glucose]), triglycerides, high-density lipoprotein (HDL) cholesterol, leptin, and hemoglobin A1c (HbA1c). The analyses of these parameters have been previously described [6]. The homeostasis model assessment-insulin resistance was used to evaluate the systemic insulin resistance index using the formula by Matthews et al.: fasting glucose (mg/dL) × fasting insulin (IU/mL)/405 [17].

### 2.8. Statistical Analysis

Unpaired *t*-tests were used to evaluate baseline differences between groups. Changes in variables from baseline to postintervention were determined by two-way time (baseline and postintervention) by-group (CR and CR + Ex) repeated measures analysis of variance. Within-group differences (i.e., baseline versus postintervention) were assessed by paired *t*-tests. In addition, because the age of the CR group was significantly younger than CR + Ex at baseline, changes in body composition (BW, FM, %FAT, VFA, and SFA), liver fat, and liver enzymes between groups were compared using analysis of covariance (ANCOVA) adjusted for age. hsCRP was logarithmically transformed to approximate normal distribution. Values of *P* < 0.05 were considered statistically significant. Data are expressed as means ± standard errors. SPSS version 12.0 software (SPSS Inc., Chicago, IL, USA) was used for all statistical analyses.

## 3. Results

### 3.1. Baseline Data and Intervention Adherences

The values for the anthropometric parameters, liver fat, blood pressure, metabolic parameters, calorie intakes, cardiorespiratory fitness, and step counts were not significantly different between the CR and CR + Ex groups at baseline. Although the CR group was significantly younger than CR + Ex group (CR; 52 ± 2 years, CR + Ex; 61 ± 2 years, *P* < 0.01), there was no correlation between age and change in liver fat. The fat intake of the CR group was significantly lower than that of the CR + Ex group (*P* < 0.05), but there was no correlation between change in fat intake and change in liver fat.

The mean percentage of supervised exercise sessions attended throughout the 12 weeks was 80 ± 4%. The average durations of exercise at home and total exercise were 139 ± 23 min/wk and 282 ± 27 min/wk, respectively. LT occurred at 57 ± 4% of VO_2peak_ and at 66 ± 3% of predicted %HR_max⁡  _ during the exercise test. The average nutritional guidance attendance rates throughout the 12 weeks were 96 ± 2% and 94 ± 2% for the CR and CR + Ex groups, respectively.

### 3.2. Effect of the Lifestyle Intervention on Changes in Body Composition, Liver Fat and Liver Enzymes, Dietary Intake, Cardiorespiratory Fitness, and Step Counts

As shown in Table 1, there were no significant group-by-time interactions for measures of body composition (BW, FM, %FAT, VFA, and SFA). There were time effects with decreases in BW, FM, %FAT, VFA, and SFA for both groups from baseline to postintervention. Changes in body composition (BW, FM, %FAT, VFA, and SFA) were not statistically significant difference between the CR and CR + Ex groups adjusted for age. There were no significant interaction effects for changes in L/S ratio, ALT, and *γ*-GTP, with similar improvements shown in both groups. There was no significant change in AST for both groups. In addition, changes in liver fat and liver enzymes were not significantly different between the CR and CR + Ex groups adjusted for age. Baseline to postintervention VO_2peak_ was at LT, and step counts increased significantly in the CR+Ex group. However, the increase in VO_2max⁡_was greater in the CR + Ex group (*n* = 10) than in the CR group (*n* = 15) (CR, −0.7 ± 0.7 mL/kg/min; CR + Ex, 2.9 ± 1.0 mL/kg/min; *P* = 0.004), and the improvement in VO_2max⁡_ was only significant in the CR + Ex group (*P* = 0.016).

### 3.3. Effects of the Lifestyle Intervention on Blood Pressure and Metabolic Parameters

As shown in Table 2, there were no significant group-by-time interactions for blood pressure, glucose, insulin, low-density lipoprotein, triglycerides, FFA, total cholesterol, hsCRP, and PAI-1. Systolic blood pressure, diastolic blood pressure, glucose, and total cholesterol were decreased from baseline to postintervention in the CR group. Triglycerides and PAI-1 were significantly reduced in the CR + Ex group. The baseline to postintervention increase in HDL cholesterol was greater in the CR + Ex group than in the CR group, and the increase was only significant in the CR + Ex group. TNF-*α* decreased significantly in the CR+Ex group.

## 4. Discussion

The results of the present study suggest that calorie restriction-induced weight loss, with or without aerobic exercise, may provide effective reductions in liver fat and liver enzymes. However, contrary to our expectations, the results of this study showed no additional reductions in liver fat and liver enzymes when aerobic exercise was prescribed together with calorie restriction. Previously, we reported that 12 weeks of aerobic exercise may reduce liver fat and liver enzymes [6]. These results were consistent with those of earlier studies where continuous aerobic exercise reduced liver fat [3–5] in the absence of weight loss. Because of these contradictory results, the effect of exercise on reducing liver fat has remained largely unclear. A few studies have showed that calorie restriction with or without exercise was an effective lifestyle modification that simultaneously reduced liver fat or liver enzymes [18–20]. Additionally, previous studies have shown that diet plus exercise-induced weight loss preferentially decreases visceral fat compared with diet alone [10, 11]. These results suggest that exercise-induced increases in catecholamines could stimulate *β*-adrenoreceptors in the VF depot, resulting in greater FFA release and oxidation [21]. The present study found no significant difference in VFA between the groups; however, VFA tended to reduce significantly in the CR + Ex group compared with the CR group (CR + Ex, −23 ± 10%; CR, −15 ± 11%, group × time for interaction; *P* = 0.075). In contrast, Ross et al. reported that when weight loss through exercise or calorie restriction is equivalent, reduction in abdominal visceral fat is similar [22, 23]. Therefore, the amount of weight change might affect most the decrease in visceral fat. In addition, the reduction in liver fat following exercise therapy alone was significant; however, the absolute change was not great compared with the approximately 10% reduction reported after an 8 kg weight loss from caloric restriction [24]. Furthermore, previous studies have revealed that aerobic exercise could reduce intrahepatic fat in the absence of weight loss [3, 25]. However, other studies have reported no such effects of aerobic exercise [26, 27]. Therefore, there is still no consensus on the effects of aerobic exercise on intrahepatic fat accumulation. The principle of decreasing liver fat is to modify lifestyle factors, such as diet and exercise, to achieve weight loss of about 10% of BW. In addition, the absolute amount of fat accumulation is much less for the liver (<200 g) compared with visceral adiposity (*≈*2 kg) or subcutaneous adiposity (*≈*20 kg) [28]. Exercise therapy appears likely to be an adjunct to caloric restriction, and the addition of moderate aerobic exercise to calorie restriction-induced weight loss might mask an effective reduction for liver fat using exercise only.

In the present study, the CR+Ex group did not lose more weight than the CR group. This result might be influenced by nonadherence to the lifestyle intervention. The subjects who achieved the target energy intake were lower in the CR+Ex group compared with the CR group (CR versus CR + Ex: 50.0% versus 13.3%, chi-square test; *P* = 0.032). Furthermore, the estimated energy expenditure attributable to the exercise in the CR + Ex group was 260 ± 127 kcal/day, which would account for a loss of fat mass of 3.0 ± 1.5 kg over 12 weeks (where 1.0 g of fat = 7.2 kcal). While there are conflicting reports [10], our results are consistent with those of earlier studies, which indicate that the magnitude of these changes is not substantially different between diet alone and diet plus exercise [29–31]. Moreover, the CR + Ex group was instructed to perform ≥300 min exercise each week; however, this ranged from 183 to 542 min. Therefore, not all of the participants in this group conducted ≥250 min exercise per week, a threshold level for clinically significant weight loss set by the ACSM [14]. Furthermore, Thomas et al. have reported that the small magnitude of weight loss observed from the majority of evaluated exercise interventions is primarily the result of low doses of prescribed exercise energy expenditures compounded by a concomitant increase in caloric intake [32]. This result might indicate that the additional effects of diet and exercise are difficult to anticipate, as suggested by the results of earlier studies [29–31].

This study revealed that there was a significant decrease in TNF-*α* in the CR + Ex group compared with the CR group. TNF-*α* is associated with fat accumulation, particularly NAFLD, and an overexpression of TNF-*α* mRNA is found in the liver and the adipose tissue of nonalcoholic steatohepatitis patients [33]. Additionally, Lambert et al. found that exercise significantly decreased TNF-*α* mRNA while weight loss had no effect, although the serum levels of TNF-*α* were not significantly altered by weight loss or exercise [34]. Furthermore, research clearly demonstrated that lifestyle interventions of diet and/or exercise had no effect on serum TNF-*α* levels in obese individuals with or without type 2 diabetes [35, 36]. Further studies are needed to elucidate the relationship between the health benefits of diet-induced weight loss with or without exercise and changes in TNF-*α*.

Earlier studies have shown a significant correlation between total fat and saturated fat intake and liver fat. In addition, Straznicky et al. underscored the important contribution of dietary factors, particularly saturated fat to liver function (ALT) [18]. However, the present study found no significant correlation between total fat and saturated fat intake and liver fat. In addition, Donnelly et al. reported that dietary fatty acids did not contribute significantly to the sources of liver triglycerides in subjects with NAFLD (dietary fatty acids 15%, serum nonesterified fatty acids 59%, and* de novo* lipogenesis 26%) [37]. In addition, the results of the present study may reflect an influence of the Japanese diet that, compared with the Western diet, is lower in total and saturated fat [38].

The present study found that cardiorespiratory fitness increased significantly in the CR + Ex group only. Furthermore, HDL cholesterol significantly increased in the CR + Ex group compared with the CR group. Exercise is a widely recognized modality for raising plasma HDL cholesterol levels and is one of the metabolic adaptations contributing to a reduced risk of coronary heart disease [39, 40]. Regular endurance exercise is particularly helpful to improve the lipid lipoprotein profile in subjects with low HDL cholesterol levels together with abdominal obesity [40]. Therefore, results of the present study indicate that calorie restriction-induced weight loss with the addition of continuous aerobic exercise causes an increase in HDL cholesterol, thereby reducing the risk of coronary heart disease.

This study had some limitations. First, we were unable to use a noninvasive and highly accurate method of identifying the amount of fat in the liver such as that achieved through using MRS. However, it has been shown that liver fat measured by CT correlates well with liver fat obtained through needle biopsy [41]. Second, all of the participants in the present study did not necessarily have intrahepatic fat accumulation. Nevertheless, the study demonstrated that calorie restriction-induced weight loss, with or without aerobic exercise, contributed to improvements in liver fat and liver enzymes. In the future, a study needs to be conducted on patients with NAFLD and NASH.

## 5. Conclusions

Calorie restriction-induced weight loss, with or without aerobic exercise, may provide an effective reduction in liver fat and arteriosclerosis risk factors in adults with visceral adiposity. In addition, the results demonstrated that there were no additional benefits relating to liver fat and liver enzymes when aerobic exercise was prescribed together with calorie restriction.

## Figures and Tables

**Table 1:** Changes in diet-induced weight loss with or without aerobic exercise on anthropometric parameter, dietary intake, cardiorespiratory fitness, and step counts.

	CR (*n* = 18)		CR + Ex (*n* = 15)			*P* value
	Before	After	Before	After	*P* value^¶^	group × time
	Av ± SEM	Av ± SEM	Av ± SEM	Av ± SEM		interaction
Age (yrs)	52 ± 2		61 ± 2		<0.001	—
Anthropometric parameter						
Height (cm)	158.2 ± 7.3	158.4 ± 7.5	158.3 ± 6.3	158.3 ± 6.2	0.958	0.354
Weight (kg)	71.3 ± 3.2	65.9 ± 2.7***	68.6 ± 3.0	63.6 ± 2.8***	0.557	0.786
Body mass index (kg/m^2^)	28.4 ± 1.0	26.2 ± 0.9***	27.3 ± 1.1	25.3 ± 1.0***	0.481	0.740
Percent body fat (%)	35.6 ± 1.8	31.3 ± 1.8***	35.5 ± 1.5	31.6 ± 1.5***	0.975	0.707
Body fat mass (kg)	25.9 ± 2.0	21.0 ± 1.6***	24.6 ± 1.6	20.2 ± 1.4***	0.617	0.635
Lean body mass (kg)	46.1 ± 2.0	45.6 ± 2.0	44.4 ± 2.2	43.4 ± 2.0*	0.556	0.537
Visceral fat area (cm^2^)	164 ± 10	139 ± 10***	165 ± 10	127 ± 10***	0.953	0.075
Subcutaneous fat area (cm^2^)	321 ± 28	282 ± 25***	287 ± 21	250 ± 22***	0.364	0.900
Liver-to-spleen ratio (HU/HU)	1.11 ± 0.09	1.23 ± 0.05*	1.15 ± 0.05	1.28 ± 0.03**	0.681	0.984
Dietary intake						
Energy intake (kcal/day)^†^	1820 ± 89	1429 ± 76***	2051 ± 83	1704 ± 75***	0.068	0.658
Protein (g/day)^†^	70.8 ± 4.8	57.9 ± 2.4**	82.7 ± 3.9	71.3 ± 4.6**	0.069	0.759
Fat (g/day)^†^	51.4 ± 3.1	39.3 ± 3.3**	64.0 ± 3.7	48.6 ± 2.9**	0.014	0.550
Carbohydrate (g/day)^†^	260.5 ± 11.8	207.6 ± 12.2***	279.3 ± 12.4	241.8 ± 10.3**	0.279	0.273
Cardiorespiratory fitness and step counts						
VO_2peak_/kg (mL/min/kg weight)^††^	23.0 ± 1.0	24.1 ± 2.2	20.7 ± 1.2	23.8 ± 1.3*	0.139	0.444
Watts at lactate threshold (watts)^ †††^	53 ± 4	53 ± 4	49 ± 4	58 ± 3*	0.432	0.053
Step counts (counts/day)^††††^	6458 ± 654	7578 ± 763	7124 ± 849	9412 ± 905**	0.536	0.257

CR: calorie restriction-induced weight loss; CR + Ex: calorie restriction-induced weight loss with aerobic exercise.

Before versus after; *P < 0.05, **P < 0.01, and ***P < 0.001.

^¶^ *P* value for baseline characteristics (CR group versus CR + Ex group).

^†^CR (*n* = 17); ^††^CR + Ex (*n* = 14); ^†††^CR (*n* = 14); ^††††^CR (*n* = 15); CR + Ex (*n* = 14).

**Table 2:** Changes in diet-induced weight loss with or without aerobic exercise on blood pressure and metabolic parameters.

	CR (*n* = 18)		CR + Ex (*n* = 15)			*P* value
	Before	After	Before	After	*P* value^¶^	group × time
	Av ± SEM	Av ± SEM	Av ± SEM	Av ± SEM		interaction
Blood pressure						
Systolic blood pressure (mmHg)^†^	136 ± 5	124 ± 4**	130 ± 3	123 ± 4	0.350	0.368
Diastolic blood pressure (mmHg)^†^	85 ± 3	78 ± 3*	81 ± 2	77 ± 3	0.287	0.398
Metabolic parameter						
Glucose (mg/dL)	99 ± 2	95 ± 2*	104 ± 4	99 ± 2	0.179	0.678
Insulin (*μ*IU/mL)	8.2 ± 0.7	6.5 ± 0.5*	9.6 ± 1.4	6.5 ± 0.9**	0.371	0.198
HOMA-IR	2.0 ± 0.8	1.5 ± 0.5*	2.6 ± 1.8	1.6 ± 0.9**		0.151
OGTT_120min_-Glucose (mg/dL)	140 ± 7	129 ± 6	165 ± 17	145 ± 12	0.142	0.494
Hemoglobin A1c (%)	5.2 ± 0.1	5.1 ± 0.1	5.5 ± 0.1	5.3 ± 0.1**	0.019	0.029
HDL (mg/dL)	51 ± 3	49 ± 2	49 ± 2	54 ± 2**	0.601	0.004
Low density lipoprotein (mg/dL)	140 ± 10	129 ± 8	126 ± 10	115 ± 8	0.352	0.977
Triglyceride (mg/dL)	145 ± 17	119 ± 17	128 ± 13	84 ± 6**	0.463	0.311
Free fatty acid (*μ*Eq/L)	303 ± 61	276 ± 55	269 ± 60	237 ± 53	0.701	0.905
Total cholesterol (mg/dL)	227 ± 12	208 ± 10*	207 ± 12	194 ± 9	0.263	0.509
Interleukin-6 (pg/mL)^††^	2.6 ± 0.8	1.2 ± 0.1	3.1 ± 0.9	2.2 ± 1.1	0.696	0.625
TNF-*α* (pg/mL)^†^	1.1 ± 0.1	1.1 ± 0.1	1.3 ± 0.2	1.1 ± 0.1*	0.130	0.027
Leptin (ng/mL)	14.6 ± 1.9	10.3 ± 1.8***	11.4 ± 1.3	6.4 ± 0.8***	0.205	0.541
High-sensitive CRP (ng/mL)^#^	2.9 ± 0.1	2.5 ± 0.1***	2.9 ± 0.1	2.6 ± 0.1**	0.864	0.537
HMW adiponectin (*μ*g/mL)	5.1 ± 0.7	5.4 ± 0.8	5.2 ± 0.7	6.0 ± 0.7*	0.861	0.186
PAI-1 (ng/mL)^†††^	29.5 ± 3.1	24.3 ± 4.1	32.8 ± 5.2	17.8 ± 2.1**	0.571	0.101

CR: calorie restriction-induced weight loss; CR + Ex: calorie restriction-induced weight loss with aerobic exercise; OGTT: oral glucose tolerance test; HDL: high-density lipoprotein; LDL: low-density lipoprotein; TNF-*α*: tumor necrosis factor-*α*; hsCRP: high-sensitive CRP; HMW: high-molecular-weight; PAI-1: plasminogen activator inhibitor-1.

Before versus after; *P < 0.05, **P < 0.01, and ***P < 0.001.

^¶^ *P* value for baseline characteristics (CR group versus CR + Ex group).

^#^High-sensitive CRP was logarithmically transformed to approximate a normal distribution.

^†^CR (*n* = 16), ^††^CR (*n* = 17); ^†††^CR (*n* = 17), CR + Ex (*n* = 13).
